# No evidence of a prospective relationship between serum zinc and venous thromboembolism in Caucasian men: a cohort study

**DOI:** 10.1007/s10534-022-00402-8

**Published:** 2022-06-10

**Authors:** Setor K. Kunutsor, Sae Young Jae, Jari A. Laukkanen

**Affiliations:** 1 National Institute for Health Research Bristol Biomedical Research Centre, University Hospitals Bristol and Weston NHS Foundation Trust and the University of Bristol, Bristol, UK; 2 Translational Health Sciences, Bristol Medical School, Southmead Hospital, University of Bristol, Learning & Research Building (Level 1), Bristol, BS10 5NB UK; 3 Diabetes Research Centre, Leicester General Hospital, University of Leicester, Gwendolen Road, Leicester, LE5 4WP UK; 4 Department of Medicine, Central Finland Health Care District, Jyväskylä, Finland; 5 Department of Sport Science, University of Seoul, Seoul, Republic of Korea; 6 Institute of Clinical Medicine, Department of Medicine, University of Eastern Finland, Kuopio, Finland; 7 Institute of Public Health and Clinical Nutrition, University of Eastern Finland, Kuopio, Finland

**Keywords:** Serum zinc, Venous thromboembolism, Risk factor, Cohort study

## Abstract

Serum zinc has been implicated as an important mediator of haemostasis and thrombosis. However, the nature and magnitude of any potential relationship between serum zinc and venous thromboembolism (VTE) is unknown. We aimed to evaluate the prospective association between serum zinc and VTE risk. We analyzed data involving 2472 men aged 42–61 years without a history of VTE in the Kuopio Ischemic Heart Disease population-based cohort study, with the assessment of serum zinc concentrations using atomic absorption spectrometry. Hazard ratios (95% confidence intervals [CIs]) for incident VTE were estimated. A total of 166 VTE cases occurred during a median follow-up of 27.1 years. The risk of VTE per 1 standard deviation increase in serum zinc in analysis adjusted for systolic blood pressure, body mass index, total cholesterol, triglycerides, smoking status, histories of type 2 diabetes and coronary heart disease, medication for dyslipidaemia, alcohol consumption, physical activity, and socioeconomic status was (HR 1.03; 95% CI 0.86–1.22), which remained similar (HR 1.04; 95% CI 0.87–1.23) following further adjustment for inflammation and history of cancer. Comparing the extreme tertiles of serum zinc, the corresponding adjusted HRs (95% CIs) were 0.92 (0.63–1.36) and 0.94 (0.64–1.39), respectively. Imputed results based on 2682 participants and 176 VTE events were consistent with the observed results. In middle-aged and older Finnish men, serum zinc is not associated with future VTE risk. Other large-scale prospective studies conducted in other populations are needed to confirm or refute these findings.

**Supplementary Information:**

The online version contains supplementary material available at 10.1007/s10534-022-00402-8.

## Introduction

Cardiovascular disease (CVD) persists as the leading cause of death globally despite major advances in the development and implementation of preventive and management strategies (Barquera et al. 2015). Cardiovascular diseases are also associated with substantial morbidity and costs to healthcare systems and economies. Atherosclerotic CVDs (arterial thrombotic disease), which include coronary heart disease (CHD) and cerebrovascular disease (ischemic stroke) (Barquera et al. 2015) are the major manifestations of CVD. Atherosclerotic CVD is closely related to venous thromboembolism (VTE), a vascular condition which comprises deep vein thrombosis (DVT) and pulmonary embolism (PE)). Both disease entities share mechanistic pathways such as coagulation, platelet activation and dyslipidaemia (Ray 2003) and common risk factors including age, obesity and cigarette smoking (Ageno et al. 2008; Glynn and Rosner 2005). Venous thromboembolism is also associated with significant morbidity, high economic costs and is a preventable cause of death (Cohen et al. 2007; Douketis et al. 2007).

Zinc, the second most abundant trace metal in the body, is an essential micronutrient that is involved in several cellular processes. It is involved in nucleic acid synthesis, enzymatic reactions, cell replication and repair, and also plays an important role in energy producing functions (Chimienti 2013). Zinc has antioxidant and anti-inflammatory properties (Jarosz et al. 2017; Olechnowicz et al. 2018). Zinc deficiency leads to many disorders. Evidence indicates that circulating levels of zinc may be involved in regulation of blood pressure (Tubek 2007) and also exhibit cardio-protective effects. Observational cohort studies have demonstrated associations between serum zinc and risk of hypertension and CVD (Kok et al. 1988; Kunutsor and Laukkanen 2016; Reunanen et al. 1996). There is mounting evidence that zinc is an important mediator of haemostasis and thrombosis. Zinc binds numerous plasma proteins and modulates their structure and function, and its deficiency is associated with bleeding and clotting abnormalities (Vu et al. 2013). Zinc is an endogenous and exogenous regulator of platelet function during haemostasis and thrombosis; extracellular zinc ions (Zn^2+^) gain access to the platelet cytosol and induce full platelet activation at high concentrations (Ahmed et al. 2021). Activated platelets also secrete zinc into the local microenvironment, with increased concentrations of zinc observed in the vicinity of a thrombus (Vu et al. 2013).

Given the linked pathways between zinc, atherosclerotic CVD, and VTE, and the biological plausibility that serum zinc may be involved in the development of VTE, we hypothesized that a potential association may exist between serum zinc and the risk of VTE. The nature and magnitude of any association between serum zinc and VTE risk using a prospective study has not been previously explored. In this context, we aimed to evaluate the prospective association between serum zinc and VTE risk using a population-based prospective cohort of 2,472 middle-aged and older Finnish men.

## Methods

Reporting of the study conforms to broad EQUATOR guidelines (Simera et al. 2010) and was conducted according to STROBE (STrengthening the Reporting of OBservational studies in Epidemiology) guidelines for reporting observational studies in epidemiology (Supplementary File 1). The Research Ethics Committee of the University of Eastern Finland approved the study (reference #:143/97), and each participant gave written informed consent. All study procedures were conducted according to the Declaration of Helsinki. Study participants were part of the Kuopio Ischemic Heart Disease (KIHD) risk factor study, a longitudinal population-based study designed to investigate risk factors for atherosclerotic CVD and other chronic diseases. Participants were a representative sample of randomly selected men living in the city of Kuopio and its surrounding rural communities in Eastern Finland. Baseline examinations were performed between 1984 and 1989 and included men 42–61 years of age. Study design and recruitment methods have been described in detail in previous reports (Kunutsor et al. 2019a, 2019b, 2020, 2018). The current analysis is based on 2472 men with no previous history of VTE and non-missing data on serum zinc, relevant covariates, and first VTE events.

The assessment of risk markers and other covariates have been described previously (Kunutsor et al. 2019a, 2019b, 2020, 2018). Besides fasting overnight, participants were told to abstain from drinking alcohol for at least 3 days and from smoking for at least 12 h before blood samples were taken between 8 and 10 a.m. Measurements of serum zinc concentrations were made from frozen serum samples stored at – 20 °C for 1–5 years. The PerkinElmer 306 atomic absorption spectrophotometer (Norwalk, Connecticut, USA) was used for the measurements, which employed a flame technique and pyrolytically coated graphite tubes with a platform (Salonen et al. 1991). Seronorm (Nycomed, Oslo, Norway) control serum samples were included in all daily batches. The reference standards were dissolved in 5% glycerol and the between-batch coefficient of variation was 4.0%. All incident VTE events that occurred from study entry to 2018 were included. All VTE events required positive imaging tests for their diagnoses and were identified by computer linkage to the National Hospital Discharge Registry data maintained by the Finnish Institute for Health and Welfare and their diagnoses. Each VTE event was validated by two physicians following detailed cross-checking of medical documents. The ICD 10 codes (I26, I80 and I82) were used to code and classify each VTE case. Hazard ratios (HRs) with 95% confidence intervals (CIs) for incident VTE were estimated using Cox proportional hazard models. The adjustment for confounders were based on four models: (Model 1) age; (Model 2) Model 1 plus systolic blood pressure (SBP), body mass index (BMI), total cholesterol, triglycerides, smoking status, history of type 2 diabetes (T2D), history of coronary heart disease (CHD), medication for dyslipidaemia, alcohol consumption, physical activity, and socioeconomic status (SES); (Model 3) Model 2 plus high sensitivity C-reactive protein (hsCRP) and history of cancer; and (Model 4) a model comprising dietary factors including serum magnesium, total energy intake, intake of processed and unprocessed red meat, and intake of fruits, berries and vegetables. The confounders selected were based on their previously established roles as risk factors for VTE, evidence from previous research, previously published associations with VTE in the KIHD study (Kunutsor et al. 2021, 2019a; Kunutsor and Laukkanen 2021), or their potential as confounders based on known associations with VTE outcomes and observed associations with serum zinc using the available data (Groenwold et al. 2011). Given the long-follow-up of the cohort, we explored the potential for regression dilution bias by conducting sensitivity analysis that was restricted to the first 10 years of follow-up. Finally, multiple imputation by chained equations (MICE) was conducted to handle potential selection bias originating from missingness. The imputation model included all model covariates as well as VTE outcome status. Ten imputations were computed due to the computational time required. Cox regression analyses were run across the 10 imputed datasets and the pooled estimates were reported. All statistical analyses were conducted using Stata version MP 16 (Stata Corp, College Station, Texas).

## Results

Table 1 shows baseline characteristics of study participants overall and by VTE development at end of follow-up. The mean [standard deviation (SD)] age and serum zinc of the 2472 men at baseline were 53 (5) years and 0.94 (0.12) mg/l, respectively. Levels of most of the risk markers including serum zinc were similar between those who developed and did not develop VTE.

**Table 1:** Baseline participant characteristics

	Overall (N = 2472) Mean (SD) or median (IQR)	No VTE (N = 2306) Mean (SD) or median (IQR)	Developed VTE (N = 166) Mean (SD) or median (IQR)
Serum zinc (mg/l)	0.94 (0.12)	0.94 (0.12)	0.94 (0.13)
*Questionnaire/Prevalent conditions*			
Age at survey (years)	53 (5)	53 (5)	54 (4)
Alcohol consumption (g/week)	31.5 (6.2–90.8)	32.0 (6.3–91.0)	29.7 (5.4–88.4)
Socioeconomic status	8.50 (4.23)	8.49 (4.22)	8.52 (4.28)
Physical activity (kj/day)	1192 (623–1991)	1189 (630–1992)	1245 (586–1908)
History of type 2 diabetes (%)	99 (4.0)	94 (4.1)	5 (3.0)
Current smoking (%)	775 (31.4)	746 (32.4)	29 (17.5)
History of CHD (%)	617 (25.0)	582 (25.2)	35 (21.1)
Medication for dyslipidemia (%)	15 (0.6)	14 (0.6)	1 (0.6)
History of cancer (%)	42 (1.7)	37 (1.6)	5 (3.0)
*Physical measurements*			
BMI (kg/m^2^)	26.9 (3.6)	26.9 (3.6)	27.2 (3.6)
SBP (mmHg)	134 (17)	134 (17)	132 (16)
DBP (mmHg)	89 (11)	89 (11)	89 (9)
*Blood-based markers*			
Total cholesterol (mmol/l)	5.91 (1.08)	5.91 (1.08)	5.91 (1.16)
HDL-C (mmol/l)	1.29 (0.30)	1.29 (0.30)	1.30 (0.30)
Serum magnesium (mg/dl)	1.98 (0.16)	1.98 (0.15)	2.00 (0.17)
Triglycerides (mmol/l)	1.11 (0.81–1.58)	1.11 (0.81–1.58)	1.19 (0.82–1.60)
High-sensitivity CRP (mg/l)	1.28 (0.71–2.44)	1.28 (0.71–1.58)	1.26 (0.67–2.38)
*Dietary intakes*			
Total energy intake (kJ/day)	9843 (2582)	9841 (2566)	9875 (2802)
Processed and unprocessed red meat (g/day)	144 (78)	144 (78)	144 (71)
Fruits, berries and vegetables (g/day)	251 (157)	251 (158)	255 (141)

*BMI* body mass index, *CHD* coronary heart disease, *CI* confidence interval, *CRP* C-reactive protein, *DBP* diastolic blood pressure, *HDL-C* high-density lipoprotein cholesterol, *IQR* interquartile range, *SD* standard deviation, *SBP* systolic blood pressure

A total of 166 VTE cases occurred during a median (interquartile range) follow-up of 27.1 (17.2–31.0) years, which corresponded to an annual rate of 2.85/1000 person-years at risk (95% CI 2.45 to 3.32). In age-adjusted analysis, the HR (95% CI) for VTE per 1 SD increase in serum zinc was 1.05 (0.89–1.24), which remained similar 1.03 (0.86–1.22) on further adjustment for SBP, BMI, total cholesterol, triglycerides, smoking status, histories of T2D and coronary heart disease (CHD), medication for dyslipidaemia, alcohol consumption, physical activity, and SES. Additional adjustment for hsCRP and history of cancer did not attenuate the association 1.04 (0.87–1.23) (Table 2). Comparing the extreme tertiles of serum zinc, the corresponding adjusted HRs (95% CIs) were 0.99 (0.68–1.45), 0.92 (0.63–1.36) and 0.94 (0.64–1.39), respectively. In the model that comprised dietary factors, the association persisted (Table 2). In sensitivity analysis restricted to the first 10 years of follow-up, the results were consistent (Supplementary File 2). Data was imputed for 2682 participants with 176 VTE events and the imputed results were consistent with those obtained using observed values (Supplementary File 3).

**Table 2:** Association between serum zinc and risk of venous thromboembolism

Zinc (mg/l)	Events/total	Model 1		Model 2		Model 3		Model 4	
		HR (95% CI)	*p-*value	HR (95% CI)	*p*-value	HR (95% CI)	*p-*value	HR (95% CI)	*p-value*
Per 1 SD increase	166/2,472	1.05 (0.89–1.24)	.55	1.03 (0.86–1.22)	.77	1.04 (0.87–1.23)	.68	1.01 (0.86–1.19)	.90
T1 (0.50–0.89)	59/904	Ref		Ref		Ref			
T2 (0.90–0.98)	56/792	1.01 (0.70–1.46)	.96	0.95 (0.66–1.38)	.80	0.97 (0.67–1.41)	.87	0.95 (0.66–1.37)	.79
T3 (0.99–1.62)	51/776	0.99 (0.68–1.45)	.98	0.92 (0.63–1.36)	.69	0.94 (0.64–1.39)	.76	0.90 (0.62–1.32)	.60

*CI* confidence interval, *HR* hazard ratio, *ref* reference, *SD* standard deviation, *T* tertile

Model 1: Adjusted for age

Model 2: Model 1 plus systolic blood pressure, body mass index, total cholesterol, triglycerides, smoking status, history of type 2 diabetes, history of coronary heart disease, medication for dyslipidaemia, alcohol consumption, physical activity, and socioeconomic status

Model 3: Model 2 plus high sensitivity C-reactive protein and history of cancer

Model 4: Serum magnesium, total energy intake, intake of processed and unprocessed red meat, and intake of fruits, berries and vegetables

## Discussion

Given the potential interplay between zinc, atherosclerotic CVD, and VTE, and that zinc may be involved in haemostasis and thrombosis, we sought to investigate the potential prospective relationship between serum zinc and VTE risk in a general population-based cohort of middle-aged and older Finnish men. Our findings showed that increased serum levels of zinc were not associated with the future risk of VTE. Results were similar in analyses restricted to the first 10 years of follow-up. Furthermore, imputed results were similar to the observed results. A detailed literature search did not identify any previous studies that have evaluated the association between zinc status and VTE. Hence, it is difficult to discuss these findings in the context of previous studies. Other large-scale studies are warranted to refute or confirm these findings.

Zinc is an essential trace element with antioxidant and anti-inflammatory activities (Jansen et al. 2009). Growing evidence derived from cell and animal studies supports a cardioprotective role of zinc. Zinc deficiency has been shown to elicit the release of pro-atherogenic factors (Reiterer et al. 2005) and supplementation with zinc has been shown to reduce atheroma formation and plasma and arterial wall lipid peroxidation and also decrease the incidence of arrhythmias (Little et al. 2010). Zinc regulates vascular endothelial cell activity (Zhu et al. 2018) and has also been implicated in arterial thrombosis (Mammadova-Bach and Braun 2019). Consistent with the mechanistic evidence, a number of observational studies have shown higher serum zinc levels or dietary zinc intakes to be associated with reduced risk of CVD (Bates et al. 2011; Kok et al. 1988; Pilz et al. 2009; Reunanen et al. 1996; Soinio et al. 2007) and better recovery of neurological deficits following an ischemic stroke (Aquilani et al. 2009). In addition to its several physiological functions, zinc has been reported to be an important mediator of haemostasis and thrombosis by modulating coagulation, platelet aggregation, anticoagulation and fibrinolysis (Vu et al. 2013). Platelet function and activation is dependent on zinc; zinc deficiency results in prolonged bleeding and reduced platelet aggregation (Mammadova-Bach and Braun 2019). Similar to calcium, zinc enhances thrombin-induced fibrin clot formation, an essential step in the haemostatic process (Mammadova-Bach and Braun 2019). It has been reported that platelet accumulation and activation at the sites of vascular injury lead to the release of zinc ions from platelets to the microenvironment of the vascular network; (Mammadova-Bach and Braun 2019) experimental evidence suggests that platelet-resident zinc may modulate the process of thrombosis, but there is uncertainty how this is achieved (Mammadova-Bach and Braun 2019).

The null findings may seem unexpected for the following reasons: (i) the close relationship between atherosclerotic CVD and VTE via shared risk factors and pathophysiological pathways and previous evidence of associations between zinc status and atherosclerotic CVD and (ii) the wealth of evidence showing that zinc may be an important co-factor in thrombosis. On the contrary, the null findings may reflect the true relationship between zinc status and future VTE risk. Zinc may represent one of the many factors involved in the thrombotic process and may not necessarily be a risk marker for VTE. These findings may also suggest important differences in the aetiology and pathophysiology of arterial thrombotic disease and VTE. Though these two disease states may be closely linked, they may in fact be two distinct diseases as viewed historically (Prandoni 2007). The evidence has not been very consistent. Whiles some studies have reported that atherosclerotic CVD is an underlying condition and precedes the development of VTE (Prandoni et al. 2003), other studies have shown that atherosclerotic CVD does not precede VTE development (Reich et al. 2006; van der Hagen et al. 2006) or VTE rather precedes atherosclerotic CVD (Prandoni et al. 2006). With regards to shared risk factors, some studies have demonstrated associations between traditional CVD risk factors and VTE risk (Ageno et al. 2008; Gregson et al. 2019), whereas, others have not (Mahmoodi et al. 2017; Wattanakit et al. 2012) Other factors that could potentially explain our null findings could be due to the population characteristics such as male only sex and the age group (middle-aged and older). The low event rate may have provided low power to detect an association. Finally, there was a potential for regression dilution bias due to availability of only single baseline measurements of zinc and the particularly long follow-up duration of the cohort, which could have underestimated the true strength of the association. (Horvei et al. 2016; Smabrekke et al. 2016) Consistent with the phenomenon of regression dilution bias, several cohort studies evaluating associations between exposures and outcomes have demonstrated significant evidence of associations at short-term follow-up, with no evidence of associations at long-term follow-up (Kunutsor et al. 2017; Quist-Paulsen et al. 2010). We attempted to explore for evidence of this bias in our cohort by restricting analysis to the first 10 years of follow-up, but the null association between serum zinc and VTE risk persisted albeit in the presence of a low event rate.

To our knowledge, this is the first study to evaluate if a temporal relationship exists between zinc status and VTE risk. Other strengths include the population-based prospective cohort design, inclusion of a random representative sample of men from an ethnically and genetically homogeneous population, the follow-up duration which was long enough for the ascertainment of VTE events, ability to adjust for a comprehensive list of risk markers for VTE, and multiple imputation to account for missing data. The limitations included inability to generalise the findings to men and other populations, lack of data on VTE subtypes and potential confounders such as history of inflammatory bowel disease and other gastrointestinal disorders, and the potential for residual confounding due to the observational design. The prolonged storage of serum samples (1–5 years) could have affected the stability of the samples. However, zinc concentrations have been shown not to be affected by prolonged storage in frozen serum samples (at – 20 ℃) for several years or repeated freeze–thaw cycles (Arnaud 2010; Pirkle 2013). We used single baseline measurements of zinc and could therefore not correct for regression dilution bias, which potentially results in the underestimation of the true association between an exposure and outcome, particularly for cohort studies with long-term follow-up (Horvei et al. 2016; Smabrekke et al. 2016). Furthermore, serum or plasma zinc concentrations may not accurately reflect actual zinc status. There is substantial interindividual variability in blood zinc concentrations with changes in dietary zinc; they respond quickly to zinc supplementation between meals than additional zinc provided in food, and are also influenced by time of the day, recent meal consumption, inflammation, hormones and certain drugs (King et al. 2015). Furthermore, given that albumin is the primary carrier protein for circulating zinc, measured zinc concentrations may not reflect actual status in some populations such as those with acute illness or malnutrition (King et al. 2015). However, given the limited data available on hair, urinary, nail, and blood cell zinc responses to changes in dietary zinc, the Biomarkers of Nutrition for Development (BOND) Zinc Expert Panel recommends the use of plasma zinc concentration as a biomarker of zinc status (King et al. 2015). Despite the many limitations and constraints of plasma zinc, it has been reported as the only biomarker of status that can be used to measure zinc status in individuals with either a low or a high supply of dietary zinc (King et al. 2015). Given that this is the first evaluation of the topic and in addition to the limitations, the findings should be interpreted with caution and should be regarded as hypothesis generating. We call on investigators with relevant data on the subject data to explore this further.

## Conclusions

Serum zinc is not associated with future VTE risk in middle-aged and older Finnish men. Other large-scale prospective studies conducted in other populations are needed to confirm or refute these findings.

## Supplementary Information

Below is the link to the electronic supplementary material.

Supplementary file1 (DOCX 42 kb)

## Data Availability

The data that support the findings of this study are available from the Principal Investigator (J.A.L.) upon reasonable request.

## Abbreviations

BMI: Body mass index
CHD: Coronary heart disease
CI: Confidence interval
CVD: Cardiovascular disease
DVT: Deep vein thrombosis
HDL-C: High-density lipoprotein cholesterol
HR: Hazard ratio
hsCRP: High-sensitivity C-reactive protein
KIHD: Kuopio ischemic heart disease
PE: Pulmonary embolism
SD: Standard deviation
VTE: Venous thromboembolism
