# Supplementary material for: No evidence of a prospective relationship between serum zinc and venous thromboembolism in Caucasian men: a cohort study
Source: Biometals. 2022 Jun 10;35(4):785–93. doi: 10.1007/s10534-022-00402-8 (PMC9314286; doi:10.1007/s10534-022-00402-8)
Supplement: Supplementary file 1 — Supplementary file1 (DOCX 42 kb) [file 10534_2022_402_MOESM1_ESM.docx]

**No evidence of a prospective relationship between serum zinc and venous thromboembolism in Caucasian men: A cohort study**

Setor K. Kunutsor^a,b,c,d,*^ Sae Young Jae^e^, Jari A. Laukkanen^d,f,g^

^a^National Institute for Health Research Bristol Biomedical Research Centre, University Hospitals Bristol and Weston NHS Foundation Trust and the University of Bristol, Bristol, UK

^b^Translational Health Sciences, Bristol Medical School, University of Bristol, Learning & Research

Building (Level 1), Southmead Hospital, Bristol, UK

^c^Diabetes Research Centre, University of Leicester, Leicester General Hospital, Gwendolen Road, Leicester, LE5 4WP, UK

^d^Central Finland Health Care District, Department of Medicine, Jyväskylä, Finland

^e^ Department of Sport Science, University of Seoul, Seoul, Republic of Korea

^f^Institute of Clinical Medicine, Department of Medicine, University of Eastern Finland, Kuopio, Finland

^g^Institute of Public Health and Clinical Nutrition, University of Eastern Finland, Kuopio, Finland

*Corresponding author at: Translational Health Sciences, Bristol Medical School, University of Bristol, Learning & Research Building (Level 1), Southmead Hospital, Bristol, BS10 5NB, UK; Phone: +44-7539589186; Fax: +44-1174147924

*Email address:* [skk31@cantab.net](mailto:skk31@cantab.net)

**Supplementary File 1:** STROBE 2007 Statement—Checklist of items that should be included in reports of cohort studies

| **Section/Topic** | Item # | Recommendation | Reported on page # |
| --- | --- | --- | --- |
| **Title and abstract** | 1 | (*a*) Indicate the study’s design with a commonly used term in the title or the abstract | Page 1 |
|  |  | (*b*) Provide in the abstract an informative and balanced summary of what was done and what was found | Page 2 |
| Introduction | | |  |
| Background/rationale | 2 | Explain the scientific background and rationale for the investigation being reported | Pages 3-4 |
| Objectives | 3 | State specific objectives, including any prespecified hypotheses | Page 4 |
| Methods | | |  |
| Study design | 4 | Present key elements of study design early in the paper | Patients and methods |
| Setting | 5 | Describe the setting, locations, and relevant dates, including periods of recruitment, exposure, follow-up, and data collection | Patients and methods |
| Participants | 6 | (*a*) Give the eligibility criteria, and the sources and methods of selection of participants. Describe methods of follow-up | Patients and methods |
|  |  | (*b*) For matched studies, give matching criteria and number of exposed and unexposed | Not applicable |
| Variables | 7 | Clearly define all outcomes, exposures, predictors, potential confounders, and effect modifiers. Give diagnostic criteria, if applicable | Patients and methods |
| Data sources/ measurement | 8* | For each variable of interest, give sources of data and details of methods of assessment (measurement). Describe comparability of assessment methods if there is more than one group | Patients and methods |
| Bias | 9 | Describe any efforts to address potential sources of bias | Patients and methods |
| Study size | 10 | Explain how the study size was arrived at | Patients and methods |
| Quantitative variables | 11 | Explain how quantitative variables were handled in the analyses. If applicable, describe which groupings were chosen and why | Patients and methods |
| Statistical methods | 12 | (*a*) Describe all statistical methods, including those used to control for confounding | Patients and methods |
|  |  | (*b*) Describe any methods used to examine subgroups and interactions | Patients and methods |
|  |  | (*c*) Explain how missing data were addressed | Not applicable |
|  |  | (*d*) If applicable, explain how loss to follow-up was addressed | Not applicable |
|  |  | (*e*) Describe any sensitivity analyses | Patients and methods |
| Results | | |  |
| Participants | 13* | (a) Report numbers of individuals at each stage of study—eg numbers potentially eligible, examined for eligibility, confirmed eligible, included in the study, completing follow-up, and analysed | Patients and methods |
|  |  | (b) Give reasons for non-participation at each stage | Patients and methods |
|  |  | (c) Consider use of a flow diagram |  |
| Descriptive data | 14* | (a) Give characteristics of study participants (eg demographic, clinical, social) and information on exposures and potential confounders | Results; Table 1 |
|  |  | (b) Indicate number of participants with missing data for each variable of interest |  |
|  |  | (c) Summarise follow-up time (eg, average and total amount) | Results |
| Outcome data | 15* | Report numbers of outcome events or summary measures over time | Results |
| Main results | 16 | (*a*) Give unadjusted estimates and, if applicable, confounder-adjusted estimates and their precision (eg, 95% confidence interval). Make clear which confounders were adjusted for and why they were included | Results; Table 2; |
|  |  | (*b*) Report category boundaries when continuous variables were categorized | Results; Table 2 |
|  |  | (*c*) If relevant, consider translating estimates of relative risk into absolute risk for a meaningful time period |  |
| Other analyses | 17 | Report other analyses done—eg analyses of subgroups and interactions, and sensitivity analyses | Supplementary Files 2-3 |
| Discussion |  |  |  |
| Key results | 18 | Summarise key results with reference to study objectives | Discussion |
| **Limitations** |  |  |  |
| Interpretation | 20 | Give a cautious overall interpretation of results considering objectives, limitations, multiplicity of analyses, results from similar studies, and other relevant evidence | Discussion |
| Generalisability | 21 | Discuss the generalisability (external validity) of the study results | Discussion |
| Other information |  |  |  |
| Funding | 22 | Give the source of funding and the role of the funders for the present study and, if applicable, for the original study on which the present article is based | After Discussion |

**Supplementary File 2:** Association between serum zinc and risk of venous thromboembolism in analysis restricted to first 10 years of follow-up

| **Zinc (mg/l)** | **Events/**  **Total** | **Model 1** | | **Model 2** | | **Model 3** | | **Model 4** | |
| --- | --- | --- | --- | --- | --- | --- | --- | --- | --- |
|  |  | HR (95% CI) | *p-*value | HR (95% CI) | *p*-value | HR (95% CI) | *p-*value | HR (95% CI) | *p-value* |
| Per 1 SD increase | 46 / 317 | 1.11 (0.83-1.50) | .46 | 1.06 (0.75-1.49) | .75 | 1.00 (0.71-1.40) | .99 | 1.11 (0.82-1.49) | .51 |
| T1 (0.50-0.86) | 18 / 108 | ref |  | ref |  | ref |  |  |  |
| T2 (0.87-0.96) | 11 / 105 | 0.65 (0.31-1.39) | .27 | 0.60 (0.28-1.31) | .20 | 0.62 (0.28-1.34) | .22 | 0.63 (0.30-1.36) | .24 |
| T3 (0.97-1.62) | 17 / 104 | 0.92 (0.47-1.81) | .81 | 0.75 (0.36-1.54) | .43 | 0.70 (0.34-1.46) | .34 | 0.92 (0.47-1.80) | .81 |

CI, confidence interval; HR, hazard ratio; ref, reference; SD, standard deviation; T, tertile

Model 1: Adjusted for age

Model 2: Model 1 plus systolic blood pressure, body mass index, total cholesterol, triglycerides, smoking status, history of type 2 diabetes, history of coronary heart disease, medication for dyslipidaemia, alcohol consumption, physical activity, and socioeconomic status

Model 3: Model 2 plus high sensitivity C-reactive protein and history of cancer

Model 4: Serum magnesium, total energy intake, intake of processed and unprocessed red meat, and intake of fruits, berries and vegetables

**Supplementary File 3:** Imputed results of the association between serum zinc and risk of venous thromboembolism

| **Zinc (mg/l)** | **Model 1** | | **Model 2** | | **Model 3** | | **Model 4** | |
| --- | --- | --- | --- | --- | --- | --- | --- | --- |
|  | HR (95% CI) | *p-*value | HR (95% CI) | *p*-value | HR (95% CI) | *p-*value | HR (95% CI) | *p-value* |
| Per 1 SD increase | 1.03 (0.88-1.21) | .73 | 1.00 (0.84-1.19) | .99 | 1.02 (0.86-1.20) | .86 | 0.99 (0.84-1.16) | .86 |
| T1 (0.50-0.86) | ref |  | ref |  | ref |  |  |  |
| T2 (0.87-0.96) | 0.97 (0.67-1.38) | .85 | 0.92 (0.64-1.32) | .65 | 0.94 (0.65-1.35) | .73 | 0.91 (0.63-1.31) | .62 |
| T3 (0.97-1.62) | 0.97 (0.67-1.42) | .89 | 0.91 (0.61-1.34) | .62 | 0.93 (0.63-1.38) | .73 | 0.88 (0.60-1.28) | .51 |

CI, confidence interval; HR, hazard ratio; ref, reference; SD, standard deviation; T, tertile

Model 1: Adjusted for age

Model 2: Model 1 plus systolic blood pressure, body mass index, total cholesterol, triglycerides, smoking status, history of type 2 diabetes, history of coronary heart disease, medication for dyslipidaemia, alcohol consumption, physical activity, and socioeconomic status

Model 3: Model 2 plus high sensitivity C-reactive protein and history of cancer

Model 4: Serum magnesium, total energy intake, intake of processed and unprocessed red meat, and intake of fruits, berries and vegetables
